# Leveraging liquid biopsy to uncover resistance mechanisms and guide personalized immunotherapy

**DOI:** 10.1016/j.tranon.2025.102445

**Published:** 2025-06-24

**Authors:** Zeinab Dalloul, Jana Grenel Briend, Mariama Diawara, Catherine Taylor, Rodney J. Ouellette

**Affiliations:** a Atlantic Cancer Research Institute, Moncton, New Brunswick, Canada; b Department of Chemistry and Biochemistry, Université de Moncton, Moncton, New Brunswick, Canada; c Dr Georges L. Dumont University Hospital, Vitalite Health Network, Moncton, New Brunswick, Canada; d Beatrice Hunter Cancer Research Institute, Halifax, Nova Scotia, Canada

**Keywords:** Liquid biopsy, Immunotherapy, Immune checkpoint inhibitors, Extracellular vesicles, Biomarkers, Resistance

## Abstract

Cancer therapy has been revolutionized by immune checkpoint inhibitors (ICIs) that create a new paradigm among cancer immunotherapies. These agents restore the immune system capacity to fight cancer through blocking the action of major immune checkpoint proteins that suppress immune responses such as programmed cell death protein 1 (PD-1), programmed death-ligand 1 (PD-L1) and cytotoxic T-lymphocyte-associated protein 4 (CTLA-4), which tumors exploit for immune escape. Another advancement is the absence of known biomarkers involves ICIs, which can further be translated into new liquid tests like liquid biopsies, indicating the immune status of the patient. Liquid biopsy is a minimally invasive method for identifying tumor-derived components such as circulating tumor cells, cell-free DNA and extracellular vesicles (EVs) from body fluids for analysis. Researchers increasingly use liquid biopsy for biomarkers discovery and patient stratification into clinical applications. Even though immunotherapy has great advances, still there are obstacles faced while using ICIs. Many patients fail to respond to the treatment because of the heterogeneous mechanisms of resistance. Immunotherapy resistance is a dynamic interplay between tumors and their surrounding stroma. To understand this variability, humanized mice models are increasingly used for mirroring human immune responses. Such models offer insight into cancer immunotherapy when human immune system is engrafted into mice, and EV and biomarker profiles are established in those models. EVs reflect differences in tumor characteristics and the immune landscape around tumors to develop personalized strategies to enhance ICI efficacy. This may improve patient prognosis, giving reason to hope for better, more effective treatments.

## Highlights

• Immune checkpoint inhibitors can restore immune system function against cancer.
• Liquid biopsies emerge as a method for biomarker discovery and guide cancer treatment.
• Immunotherapy resistance needs the understanding of tumor-immune system interactions.
• Humanized Mouse Models offer better insights for studying cancer immunotherapy.

## Graphical abstract

**Figure:**
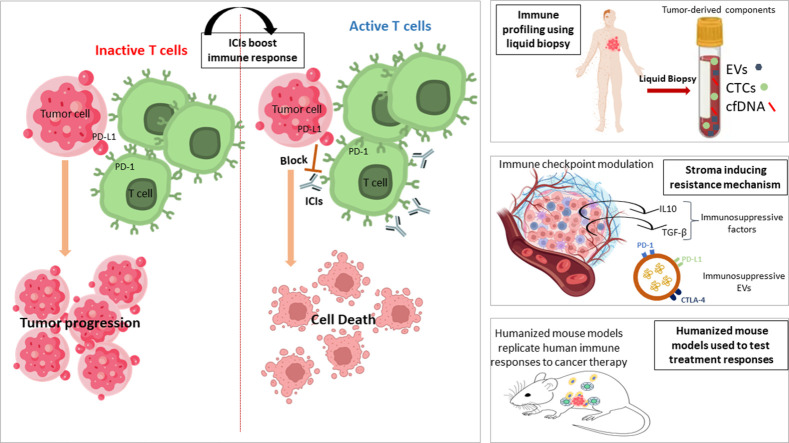

## Background

Cancer is a significant public health problem all over the world. Its prevalence underlines the urgent need for comprehensive strategies and resources in combating its devastating effects. Although emerging therapies have improved treatment options over the past decade, survival benefit remains limited to a specific subgroup of cancer patients, highlighting the ongoing challenge of making treatments effective for a wider range of cancer patients. Recent advances in our understanding of tumor immunology and the development of new cancer immunotherapies have opened a new chapter in the fight against cancer due to the discovery of cancer immune checkpoints and the success of ICI drugs (J. F. A. P [67]).

Liquid biopsy is a minimally-invasive method that facilitates the dynamic monitoring of a wide variety of circulating components which can provide real-time interrogation of the cancer and immune contexture. Liquid biopsy approaches provide a non-invasive alternative to conventional tissue biopsies through the detection of circulating tumor elements, including circulating tumor cells (CTCs), cell-free DNA, and exosomes circulating in the blood or any other body fluids. These biomarkers can carry crucial information on the genetic mutations, gene expression patterns, and other molecular features of tumors. Analyzing these biomarkers by liquid biopsy, clinicians and researchers can comprehensively understand the genetic makeup and dynamics of a patient's cancer without the need for an invasive procedure [100].

Immunotherapy has revolutionized cancer treatment by harnessing the immune system’s ability to recognize and eliminate tumor cells. Rather than directly targeting tumors, immune checkpoint inhibitors (ICIs) target checkpoint proteins and act by releasing the brakes on T cell activity. ICIs targeting CTLA-4, PD-1 and PD-L1 have dramatically improved survival rates across diverse cancer types, including non-small cell lung cancer (NSCLC), melanoma, renal cell carcinoma (RCC), hepatocellular carcinoma, and triple negative breast cancer. However, the use of ICIs can result increased incidences of immune-related adverse events (irAEs), particularly when anti-CTLA-4 and anti-PD-1 antibodies are combined, leading to potentially severe autoimmune toxicities [82]. Other cancer immunotherapy strategies have also emerged, including cancer vaccines targeting tumor-specific antigens, cytokine therapies (e.g., IL-2 and IFN-α for melanoma and renal cancer), and cell therapies, such as CAR-T, which has revolutionized the treatment of hematologic malignancies like B-cell acute lymphoblastic leukemia and multiple myeloma [58]. Immunotherapy offers a growing arsenal of treatment options for cancer with new indications and treatment combinations continuing to emerge; however, its application in personalized medicine requires careful consideration of patient-specific clinicopathological factors, predictive biomarkers and toxicity profiles for successful implementation [8,58].

Combining the advantages of liquid biopsy with translational immunotherapy research has profound implications for improving personalized cancer care. Liquid biopsy can offer valuable information on a patient's tumor profile about its mutational landscape and potential biomarkers predictive of response to this kind of therapy. In this regard, the application of liquid biopsy to identify specific genetic alterations or immune-related biomarkers present in circulating tumor components can help oncologists determine the most appropriate targeted therapy or immunotherapy regimen for individual patients to be administered for maximal treatment effect and clinical outcome improvement [4]. Yet, there are still challenges that do not go away, like resistance mechanisms and patient heterogeneity. Resistance to immunotherapy can be traced back to the ever-changing interaction between tumors and stroma. Resistance can be categorized into two types: primary resistance where patients do not respond at all to the treatment and secondary resistance where patients initially respond to treatment and treatment resistant emerges during treatment [44].

In this review, we will discuss the potential of liquid biopsy in identifying mechanisms of treatment resistance, particularly in the context of immunotherapy. We will explore the crosstalk between liquid biopsy/biomarkers and immunotherapy, to understand why some patients are responder to immunotherapy and why some others are non-responders. Although we discuss in depth the complex resistance mechanisms to immunotherapy, we explore further new possibilities, including extracellular vesicles as dynamic information reservoirs in the context of treatment resistance. Finally, we discuss the role of humanized mouse models in unraveling the delicate balances of immunotherapeutic approaches, and a foretaste of their potential transfer of paradigm in fine-tuning treatment strategies.

## Immunotherapy

**Fig. 1:**
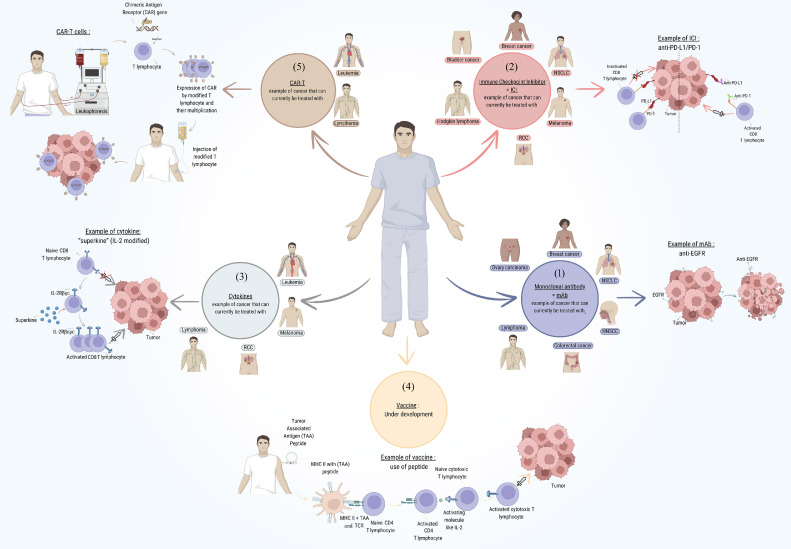
Types of immunotherapies used in cancer treatment. Schematic representation of several strategies that have been developed to take advantage of the immune system's capacity to selectively destroy cancer cells. The major immunotherapy approaches include: 1) Monoclonal Antibodies: Constructed antibodies that specifically bind either to cancer cells or to proteins of the immune system to enhance the immune response. 2) Immune checkpoint inhibitors are drugs that block proteins, such as PD-1/PD-L1 or CTLA-4, that prevent immune cells from attacking cancer cells, reactivating the immune system in the process. 3) Cytokines are signaling proteins, which include interleukins or interferons, that stimulate the immune system to attack cancer cells. 4) Cancer vaccines are designed to provoke the immune system into recognizing and attacking cancer-specific antigens. 5) CAR-T Cell Therapy: A personalized treatment wherein the patient's T cells are genetically engineered to express chimeric antigen receptors, or CARs, which let them target and destroy cancer cells more effectively.

Immunotherapy is a new and advanced way of treating cancer, using the patient's immune system to fight cancer cells. Immunotherapy is different from types of treatments like chemotherapy and radiation which are attacking the cancer cells directly -instead, it activates an immune response by removing obstacles that prevent the immune system from recognizing and killing the tumor cell. Immunotherapy has reshaped cancer treatment by enabling durable responses and prolonged disease control in some patients. Although immune checkpoint inhibitors are generally better tolerated than chemotherapy or radiotherapy, they can still cause severe immune-related side effects, particularly when used in combination. There are different types of immunotherapies used to care cancer: Monoclonal antibodies, Immune checkpoint inhibitors, Cytokines, cancer vaccines and CAR-T cell therapy (Fig. 1).

### Monoclonal antibodies

Monoclonal antibodies or MABs are glycoproteins composed from two heavy chains and two light chains connected by disulfide bonds; MABs are produced by B lymphocytes and are implicated in humoral immunity [31]. The main function of MABs is the recognition and neutralization of foreign antigens. In cancer immunotherapy, therapeutic MABS can be used in a number of different ways, including blocking growth factor receptors, activating tumor cytotoxic pathways, and activating immune-mediated that engage the patient’s immune system to eliminate tumor cells. MABs paly a critical role in cancer immunotherapy treatments and can act either by direct effect on malignant cells or by enhancing host defense systems against malignant cells [117]. By preventing ligand-receptor interactions, MABs can directly block critical tumor signaling pathways that are necessary for tumor cell proliferation and survival. For example, trastuzumab is a MAB that recognizes the HER2 receptor, effectively halting proliferative signaling in HER2-positive breast cancer cells. MABs can also induce apoptosis in tumor cells by cross-linking death receptors on tumor cells to trigger programmed cell death independent of immune cells. Antibody–drug conjugates (ADCs), in which cytotoxic agents are coupled to tumor-targeting antibodies, are another strategy that facilitates precise delivery of chemotherapy to malignant cells while minimizing systemic toxicity [65]. MABS with immune-mediated effector functions are another important class of immunotherapy. Antibody-dependent cellular cytotoxicity (ADCC) occurs when the Fc region of IgG antibodies bind to FcγRIIIa receptors on natural killer (NK), causing the release of cytotoxic granules, such as perforin and granzymes, that cause tumor lysis [117]. Another mechanism of MAB-mediated tumor cytotoxicity is Complement-dependent cytotoxicity (CDC), in which Fc-mediated recruitment of the C1q component activates the classical complement cascade, leading to the formation of the membrane attack complex and subsequent tumor cell lysis. Finally, antibody-dependent cellular phagocytosis (ADCP), wherein recognition of antibody-coated tumor cells by macrophages or dendritic cells via Fcγ receptors results in the engulfment and lysosomal degradation of the target cell [39]. Together, these various mechanisms of achieving tumor lysis underscore the versatility and potency of mAbs as a cornerstone of cancer immunotherapy.

Recent innovations to improve MABs include engineering of the Fc region for enhanced binding to Fcγ receptors, resulting in improved ADCC and ADCP responses. In addition, afucosylated antibodies, have demonstrated superior NK cell activation. Moreover, bi-specific and tri-specific antibodies that can recognize two or three targets simultaneously are under active clinical investigation, thereby expanding therapeutic versatility [29]. FDA-approved MABS are now in clinical use in multiple cancer indications, such asrituximab (anti-CD20) for CLL, cetuximab (anti-EGFR) for EGFR+ colorectal cancer, trastuzumab (anti-HER2) for HER2+ breast cancer, and elotuzumab (anti-SLAMF7) for multiple myeloma. Optimization of antibody design to improve pharmacologic properties by altering glycosylation and isotype structure, continues to be an active area of investigation [103].

#### Immune checkpoint inhibitors (ICI)

Immune checkpoints are molecules that regulate mechanisms modulating the immune system to avoid its overactivity, which can lead to autoimmune diseases. These checkpoints include proteins such as CTLA-4, PD1 and PD-L1, which prevent T-cell mediated killing of healthy cells by interaction with binding partners on activated immune cells which suppress their function. These checkpoints can, during tumorigenesis, be exploited by tumors through the expression of ligands like PD-L1, which binds to checkpoint proteins on T cells, dampening their antitumor activity and enabling the cancer to escape immune surveillance. By disabling these checkpoints, immune checkpoint inhibitors allow T cells to recognize and destroy cancer cells more effectively. This has been one of the most innovative methods in the treatment of cancer over the past few years, able to give new hope to suffering patients because it gears up the body's own defenses for fighting back against the disease more effectively.

Most used ICIs used in the last decade are monoclonal antibodies. The FDA (Food and Drug Administration) has approved several groups of ICIs to treat a variety of cancers: ipilimumab (CTLA-4 inhibitor); nivolumab, pembrolizumab, and cemiplimab (PD-1 inhibitors); atezolizumab, durvalumab, and avelumab (PD-L1 inhibitors) [54]. More recently, anti-LAG-3 antibodies, such as relatlimab, have received approval for use in combination with nivolumab in advanced melanoma [99]

***CTLA-4 inhibitors:*** Cytotoxic T-lymphocyte-associated protein 4 (CTLA-4) is an inhibitory immune checkpoint receptor that is expressed on activated T cells and regulatory T cells. CTLA-4 downregulates early immune responses by competing with CD28 for binding to CD80/CD86 on antigen-presenting cells. ICI-mediated blockade of CTLA-4 enhances T-cell activation and proliferation, particularly in lymphoid tissues. Ipilimumab, a humanized CTLA-4 monoclonal antibody, was the first ICI approved by the FDA and remains a cornerstone of cancer immunotherapy, particularly for unresectable/metastatic melanoma [23] and metastatic esophageal squamous cell carcinoma [57]. Ipilimumab is also used in combination with nivolumab (anti-PD-1), which enhances both priming and tumor infiltration of activated T cells, resulting in improved survival outcomes in melanoma, RCC, and NSCLC [14,83,113]. More recently, another anti-CTLA-4 monoclonal antibody, tremelimumab, was approved as part of the STRIDE regimen, in which a single priming dose of tremelimumab is used in combination with repeated doses of durvalumab (anti-PD-L1 antibody) to treat unresectable hepatocellular carcinoma. This approval further demonstrates the value of CTLA-4 blockade beyond melanoma and underscores the therapeutic value of dual checkpoint inhibition [1]. The reasoning behind combining CTLA-4 with PD-1/PD-L1 blockade lies in their complementary mechanisms of action: inhibition of CTLA-4 increases T-cell expansion during the initial immune priming phase, while PD-1/PD-L1 blockade re-invigorates effector T cells within the tumor microenvironment (TME). The pursuit of safer and more effective immune checkpoint blockadehas led to the development of nanobodies—antibody fragments derived from the variable domain of heavy-chain-only antibodies. Their small size and stability improve tumor penetration and mayreduce immunogenicity. While nanobody-based CTLA-4 inhibitors are currently under investigation, the use of single-chain variable fragments (scFvs) has also emerged as a promising method of improving ICI. These scFvs fragments which are composed of linked variable regions of heavy and light chains, can be incorporated into bispecific or CAR-T constructs to modulate CTLA-4 checkpoint activity. The evolving role of scFvs in the development of new immunotherapy drugs and checkpoint blockade strategies is an area of intense investigation [57]. Unfortunately, the greater treatment efficacy offered by dual blockade is accompanied by a higher incidence of immune-related adverse events, necessitating careful patient selection and dose optimization [80]. Novel applications of CTLA-4 inhibitors are under active investigation, either alone or in combination with other immunotherapies, such as dual checkpoint blockade, CAR-T enhancements, and intra-tumoral antibody delivery. These studies aim to expand the benefit of CTLA-4 blockade to a wider range of patients and indications while mitigating systemic toxicity. Notably, several clinical trials are assessing the efficacy of lower doses of ipilimumab or pairing it with new immunotherapies, such as LAG-3 antibodies, reflecting a shift toward precision combination regimens in immuno-oncology [104,108].

***PD-1/PD-L1 inhibitors:*** Programmed cell death protein 1 or PD-1 is an immune checkpoint receptor first discovered in 1992 in apoptotic T cells [35]. Later, PD-1 was also found to be expressed in other cells including B cells, MDSCs cells (myeloid-derived suppressor cells) and NK cells ([2,15,35,78]; Y [123]). PD-1 can interact with its ligand PD-L1 expressed on both antigen presenting cells (APC) and cancerous cells. PD-1/PD-L1 binding inhibits T cells cytotoxic activity and induces T cells exhaustion leading to T cells death [15,37]; Y [123]. To enhance the cytotoxic effects of T cells against cancerous cells, blocking the PD-1/PD-L1 pathway is widely employed as an immunotherapy strategy aimed to improve survival. Several anti-PD1 or anti-PD-L1 antibodies have been approved, for treatment of various types of cancer, including such as durvalumab (anti-PD-L1), nivolumab (anti-PD1) and pembrolizumab (anti-PD1), which are used to treat skin cancer, NSCLC, SCLC, HCC, bile duct cancer, endometrial cancer and certain types of breast cancer. In addition to the current PD1/PD-L1 blockade drugs that have become standard-of-care, several new PD-1 inhibitors have recently received FDA approval for certain indications. For instance, dostarlimab was approved for mismatch repair-deficient (dMMR) endometrial cancer and demonstrated complete clinical response in locally advanced dMMR rectal cancer, as demonstrated in a landmark phase II study [17]. Retifanlimab was approved for advanced Merkel cell carcinoma and is under investigation for other solid tumors. Tislelizumab, which was engineered to minimize Fcγ receptor binding and thereby improve safety, was recently approved for esophageal squamous cell carcinoma and NSCLC. Toripalimab, which was approved by the FDA in 2023 for recurrent or metastatic nasopharyngeal carcinoma demonstrated significant survival benefit in the JUPITER-02 trial [52]. PD-1/PD-L1 inhibitors promote tumor infiltrating lymphocytes (TIL) activation and inhibiting apoptosis of T cells, in addition to promoting cytotoxic effects of granular enzyme and perforin production. PD-1/PD-L1 inhibitors also increase the secretion of pro-inflammatory cytokines (e.g., IFN-γ, IL-2, TNF-α) and inhibit the secretion of immune inhibitory cytokine IL-10 [64,77], p. 27; [86]; F [91,106]. Some features of anti-PD-1 and anti-PD-L1 monoclonal antibodies, such as low tissue permeability, immune-related adverse effects, and high cost, means that better predictive biomarkers are needed so that they are only used to treat the patients most likely to benefit from alternative ways to treat cancer. Several mouse studies showed that small molecule inhibitors of PD-1 or PD-L1 also show promise as future immunotherapy treatments. Recently, Yulai Liang at al. showed that PD-L1 specific affinity peptides (PPL-C) exhibited antitumor effect in colon cancer by inhibiting PD-1/PD-L1 interaction. This study also revealed that PPL-C reduces tumor mass and enhances T lymphocytes cytotoxic effect in CT26-bearing mice [53]. Another study published in 2024 also demonstrated that a-hPD-L1 iBodies (polymer-based mimetic of anti-human PD-L1 antibodies) developed by conjugating the macrocyclic peptide WL12 to a copolymer of N-(2-hydroxypropyl) methacrylamide, inhibit PD-1/PD-L1 interaction and enhance T cells cytotoxicity. These data suggest that iBodies targeting PD-L1 could serve as a potential molecule in immunotherapy [118].

The primary problem of PD-1/PD-L1 inhibitors is the unequal response rate in overall patients. While these immunotherapies have shown remarkable success in certain individuals by activating the immune system against cancer cells, a significant number of patients do not respond. A recent study showed a difference in sex-related immune microenvironment, especially CD4+ *T* cells, in response to PD-1 blockage combined with chemotherapy for NSCLC patients. In fact, females would drive a larger benefit from PD-1 blockage than males with advanced or metastatic NSCLC [115]. Identifying other predictive biomarkers would help to distinguish patients who are likely to respond or not respond to immunotherapy.

#### Cytokines

Cytokines are small secreted immune-modulatory proteins, such as growth factors, chemokines, interleukins, interferons, and colony-stimulating factors, and were deployed as the earliest form of immunotherapy used to treat cancer. Treatment with cytokines in the oncology setting primarily involves the administration of recombinant interleukins or interferons to enhance the activity of immune effector cells, with the most common in clinical use being interleukin-2 (IL-2) and interferon-alpha (IFN-α). High doses of IL-2 for the treatment of RCC and metastatic melanoma was one of the first immunotherapies to receive FDA approval, owing to its capacity to expand and activate cytotoxic T cells and natural killer (NK) cells [10].

The use of cytokines for cancer therapy has been limited by their short half-life in circulation as well as poor tolerability and systemic toxicity which necessitates intensive patient monitoring. Innovations in engineering of recombinant cytokines are working to improve efficacy and safety of cytokine therapy. For example, modified IL-2 variants such as bempegaldesleukin (NKTR-214), a CD122-biased agonist, are being developed that preferentially stimulate effector T cells and NK cells while minimizing activation of regulatory T cells and reducing toxicity [85]. The use cytokine therapy to potentiate the anti-tumoral effects of ICIs is also an area of active investigation. Recent studies, both preclinical and early clinical trials, suggest that cytokines can remodel the TME to reduce immune suppression and improve infiltration by cytotoxic lymphocytes I, thereby improving responsiveness to ICIs [10]. Furthermore, interleukins such as IL-12 and IL-15 are under investigation for their ability to boost the persistence and anti-tumoral activity of CAR-T cell therapies, thereby extending the durability of responses in hematologic and solid tumors [94].

#### Cancer vaccines

Another type of immunotherapy is cancer vaccines which are designed to educate the immune system to recognize and attack tumor-associated antigens (TAAs) or tumor-specific antigens (TSAs). Unlike vaccines that are given prophylactically to prevent infectious diseases, cancer vaccines are typically used therapeutically to treat existing malignancies by stimulating tumor-directed immune responses. Early cancer vaccines took advantage of common TAAs, such as HER2/neu, MUC1, or WT1; however, their effectiveness was often limited by variable expression and the development of immune tolerance.

Recent advances in high-throughput genomics and bioinformatics have fostered the development of personalized approaches to cancer vaccines using neoantigens designed from patient-specific somatic mutations. Neoantigens are tumor-specifc antigens (e.g. a peptide with a tumor-specific somatic mutation) and as such are recognized as being foreign by the immune system, making them ideal targets with minimal risk of off-target effects to healthy tissue. The creation of a personalized neoantigen vaccine typically requires high-throughput sequencing of the patient’s tumor and normal DNA, followed by in silico prediction of peptide-MHC binding activity and T cell recognition. The neoantigen peptide is then delivered to the patient as either a mRNA, peptide, or dendritic cell-based vaccine.

A 2024 study of high-risk melanoma demonstrated that combining an mRNA neoantigen vaccine (mRNA-4157/V940) with pembrolizumab significantly improved recurrence-free survival compared to pembrolizumab alone [110]. The robust CD8+ *T* cell responses generated against the predicted neoepitopes in this study highlight the potential of personalized vaccines to synergize with ICI to improve patient outcomes. Similar strategies are now under investigation for lung, pancreatic, renal, and colorectal cancers.

Despite their promise, challenges in the use of cancer vaccines remain, such as variability in neoantigen prediction accuracy, delays in treatment due to vaccine production time, and tumor evolution that can lead to antigen escape. Nonetheless, the development of patient-specific neoantigen cancer vaccines represents a major advancement in precision immunotherapy and is an area of intense clinical investigation [69].

#### CAR-T cell therapy

Chimeric Antigen Receptor T-cell (CAR-T) therapy is a form of immunotherapy that employs adoptive cell transfer of T cells that have been modified to express synthetic receptors (CARs). This modification allows the T cells to that enable them to recognize and kill tumor cells independently of major histocompatibility complex (MHC) presentation. CARs are typically created using an extracellular tumor antigen-specific single-chain variable fragment (scFv), a transmembrane domain to allow insertion into the T-cell membrane, and an intracellular signaling domain to facilitate T cell activation upon antigen recognition, such as a CD3ζ and co-stimulatory molecules (e.g., CD28 or 4–1BB).To generate CAR-T cells, autologous T cells are isolated from the patient and modified ex vivo using viral or non-viral vectors to express the CAR construct. The CAR-T cells are then expanded and reintroduced into the patient following lymphodepletion. Once infused, CAR-T cells seek out and destroy cancer cells expressing the target antigen.

CAR-T therapies have been revolutionary in the treatment of hematological malignancies. As of 2024, six FDA-approved CAR-T cell products are in clinical use, including tisagenlecleucel (for B-cell acute lymphoblastic leukemia), axicabtagene ciloleucel and lisocabtagene maraleucel (for diffuse large B-cell lymphoma), brexucabtagene autoleucel (for mantle cell lymphoma), and ciltacabtagene autoleucel (for multiple myeloma) [30]. Complete and durable disease remission is often observed in CAR-T-treated patients with otherwise refractory disease.

New developments in CAR-T cell therapy are quickly expanding their use in indications other than hematologic cancers. Improvements in CAR design, such as the use of novel or multiple antigens, the use of switchable or logic-gated CARs, and the use of chemokine receptors, are among the ways researchers are addressing the challenge of overcoming tumor heterogeneity and an immunosuppressive TME to improve t cell infiltration, thereby making the use of CAR-T therapy in solid cancers possible. Additionally, off-the-shelf “allogeneic” CAR-T therapies using CRISPR or TALEN gene editing are in clinical trials to overcome limitations of patient-specific manufacturing and to improve accessibility [60].

Despite their success, the use of CAR-T therapies is sometimes accompanied by significant toxicities, including cytokine release syndrome (CRS) and immune effector cell-associated neurotoxicity syndrome (ICANS), which require specialized management. To reduce these undesirable adverse events, therapeutic regimens with better tolerability and biomarker-driven patient selection are in development.

### Tumor microenvironment (TME), resistance to ICI therapy and immunosuppressive factors

**Fig. 2:**
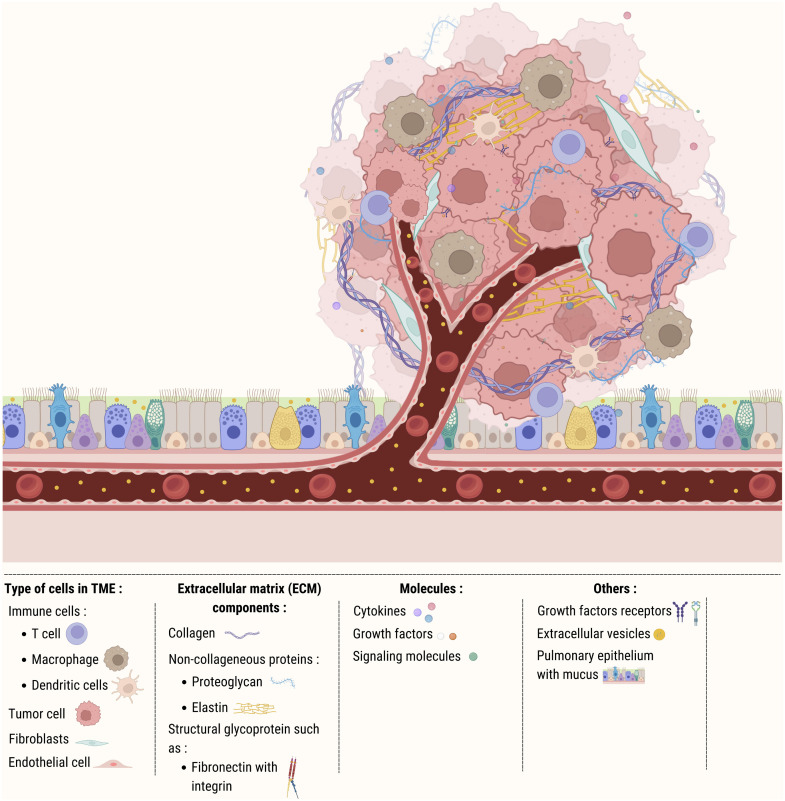
Principal immune cells and components of the tumor microenvironment. This figure depicts some of the main cellular and molecular players in the TME, including immune cells like T cells, macrophages, and dendritic cells, but also non-immune cells such as fibroblasts and endothelial cells. It also illustrates the role of various extracellular matrix components, including collagen, non-collagenous proteins, glycoproteins, cytokines, growth factors, and signaling molecules in tumor progression, immune evasion, and therapeutic response.

The development of resistance to ICIs during cancer treatment poses a significant challenge and is influenced by the tumor microenvironment (TME). The TME is a complex, dynamic ecosystem comprising a diverse array of cellular and non-cellular elements interacting intricately with cancer cells to drive tumor growth, progression, and response to treatment. Key immune cells in the TME include T cells, macrophages, and dendritic cells, alongside fibroblasts, endothelial cells, and extracellular matrix (ECM) components such as collagen, non-collagenous proteins, glycoproteins, cytokines, growth factors, and signaling molecules (Fig. 2).

Immune cells within the TME play dual roles; they can either promote tumor growth via immunosuppressive mechanisms or inhibit tumor growth through enhanced immune surveillance and cytotoxic responses. For instance, regulatory T cells (Tregs) and myeloid-derived suppressor cells (MDSCs) create an immunosuppressive environment that shields tumors from immune attack [20,55,62,63], whereas cytotoxic T cells and natural killer (NK) cells exert anti-tumor effects [71,114]. Fibroblasts contribute to ECM remodeling and produce growth factors that support tumor growth and metastasis [45]. Resistance to ICI can be, in part, attributed to the presence of M2 macrophages in the TME, where they secrete immunosuppressive cytokines (e.g., IL-10, TGF-β), promote angiogenesis, and inhibit cytotoxic T lymphocyte activity, thereby facilitating immune escape and tumor progression. Infiltration of M2 macrophages into the TME is associated with poor response to PD-1/PD-L1 blockade and reduced overall survival [16,49]. Therefore, targeting M2 macrophage polarization, M2 recruitment, or strategies to reprogram them toward an M1-like pro-inflammatory phenotype is an emerging strategy to overcome resistance and enhance immunotherapy efficacy. Combining the use of ICIs and drugs that modulate macrophage polarization, such as colony stimulating factor 1 receptor inhibitors, are currently under investigation in preclinical and clinical oncology studies. Angiogenesis, driven by factors produced by both tumor and stromal cells, supplies essential nutrients and oxygen, shaping the TME and influencing tumor behavior [112]. Cytokines and growth factors secreted within the TME further facilitate cell communication, angiogenesis, tumor growth, and modulation of immune responses. Targeting the TME has shown therapeutic potential in cancer, with strategies such as immune checkpoint inhibitors (ICIs) [119] and CAR T-cell therapy [27,38] enhancing anti-tumor immunity by overcoming immunosuppressive mechanisms within the TME.

Resistance mechanisms include inadequate antigen recognition by T cells, impaired T-cell migration and infiltration, and decreased T-cell cytotoxicity, often linked to the T-cell activation pathway. Antigen-presenting cells (APCs) present cancer antigens to T cells, initiating activation pathways that enable T cells to target cancer cells. Defects in antigen recognition disrupt this process, weakening anti-tumor immunity. Patients whose tumors have a high tumor mutational burden (TMB) often respond well to ICIs due to strong T-cell activation by cancer neoantigens [70], but changes in these neoantigens or their loss can create resistance [5,87]. Additionally, defective APC recruitment to the TME or downregulation of major histocompatibility complex (MHC) molecules by cancer cells due to genetic or epigenetic abnormalities also reduces antigen presentation and anti-tumor immunity [70].

Resistance to ICI therapy can be categorized as primary, adaptive, or acquired. Primary resistance involves pre-existing tumor characteristics that confer resistance, such as mutations disrupting antigen presentation, dysregulation of immune checkpoints, down modulation of T-cell recognition elements, or production of immunosuppressive factors within the TME [7]. Adaptive resistance refers to changes in tumors during treatment, enabling them to evade immune responses by altering the expression of immune-related molecules and pathways [25]. Acquired resistance occurs when a tumor initially responds to immunotherapy but later relapses, often due to genetic or epigenetic changes or clonal evolution within the cancer cell population [96].

Tumors employ various mechanisms to evade immune detection and destruction, such as recruiting regulatory T cells, myeloid-derived suppressor cells, and tumor-associated macrophages [101], which suppress cytotoxic T cells and NK cells. Tumor cells may also upregulate immune checkpoint molecules like PD-L1, which binds to inhibitory receptors on T cells, leading to immune tolerance [11,79]. Additionally, tumors secrete factors like TGF-β, which inhibits T cell and NK cell responses [56,111], and IL-10, which is elevated in various cancers including NSCLC, pancreatic cancer, leukemia, and squamous cell carcinomas of the head and neck [90]. The tumor coagulome also facilitates immune evasion by creating inflammation and recruiting platelets, neutrophils, and macrophages, leading to immunothrombotic reactions [107]. Prostaglandin E2 (PGE2), produced by tumor cells and immune cells, modulates immune responses by inhibiting cytotoxic T lymphocytes and promoting immunosuppressive cells [12].

## Liquid biopsy for cancer biomarkers of response

Up to now, tissue biopsy is considered one of the most conventional approaches used for cancer diagnosis [51]. Even if tissue biopsy is informative, it is a painful procedure, invasive and sometimes inappropriate for capturing tumor heterogeneity and for following tumor progression [88]. On the other hand, there are no reliable biomarkers capable of accurately predicting ICI treatment response or which allow clinicians to monitor tumor response. Such a treatment monitoring approach would open the pathway to liquid-based biomarker, providing more precise information on changing immune profiles of patients. By tapping into ICIs, an opportunity is presented to establish novel sources of biomarkers to not only improve diagnostic precision but also to deepen our understanding of immune responses in patients. Cancer liquid biopsy, coined in 2010, is a revolutionary strategy characterized by the non-invasive detection of cancers from biofluids like blood, saliva and urine for cancer diagnosis, prognosis and for monitor real-time cancer evolution [3]. In the clinic, blood is readily available for liquid biopsy approaches. Presently, cancer liquid biopsy analysis tends to focus on three main types of circulating biomarkers: circulating tumor cells (CTCs), extracellular vesicles (EVs) and cell-free nucleic acids which are derivatives of primary and metastatic sites [3].

### Circulating tumor cells

Circulating tumor cells are cells detached from the primary tumor or established distant sites to invade the bloodstream through a process called intravasation. These cells can re-establish themselves at distant sites through a process called extravasation. During their journey in the blood, CTCs interacts with blood cells, such as platelets and immune cells. Platelet-derived TGF-β and the direct contact of platelets with CTCs activate the TGFβ/Smad and NF-κB pathways in tumor cells and enhance metastasis in vivo [48]. Additionally, it was shown that platelet aggregation with CTCs protects them from the cytolysis effects of NK killer cells, thereby supporting the CTCs and promoting the metastasis process [72]. CTCs also interact with immune cells like neutrophils [98], myeloid cells [59] and cancer-associated fibroblasts [24] and thereby make CTCs dissemination easier.

CTCs in the bloodstream can avoid immune system attacks by several mechanisms. In 2005, Nicholas F.S. Watson [109] showed that colorectal tumor cells downregulate surface MHC class I molecules and evade from T-cell-mediated attack. Additionally, to escape from programmed cell death (apoptosis), CTCs express high levels of FAS ligand (FAS-L or CD95-L) [33], a transmembrane protein expressed on several types of cells, including cytotoxic T lymphocytes, monocytes, neutrophils and natural killer (NK) cells. Interaction of FAS-L expressed on CTCs surface with FAS expressed on the surface of cytotoxic T cells (CD8+ *T* cells) leads to T cells apoptosis [74].

### Extracellular vesicles (EVs)

**Fig. 3:**
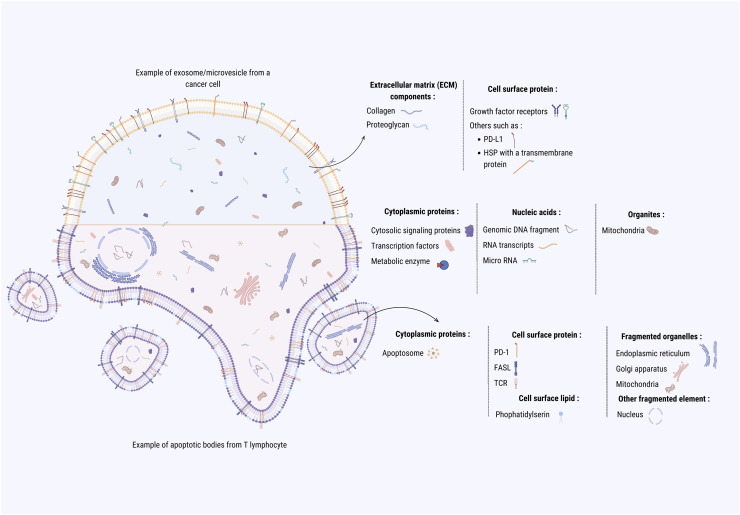
Types and Composition of Extracellular Vesicles (EVs). Extracellular vesicles are the lipid membrane-bound structures mediating cell-to-cell communication by transporting proteins, nucleic acids, and lipids. They are heterogeneous in size and composition. EVs contain proteins, lipids, and RNA, which reflect the cell of origin.

Extracellular vesicles are tiny, lipid membrane-bound structures released by cells into the extracellular space [116]. These vesicles enable cell-to-cell communication by facilitating the trafficking of various molecules, such as proteins, nucleic acids, and lipids, which reflect the cell of origin [9]. EVs are heterogeneous in size, composition, and function and include several subtypes, including exosomes, which are small particles of endosomal origin (typically 50–150 nm), microvesicles which vary in size from 50–1000 nm and result from budding of the plasma membrane, and large (>1000 nm) apoptotic bodies (Fig. 3) [21]. Exosome biogenesis involves the invagination of the endosome membrane, forming a multivesicular endosome. Subsequently, the structures fuse with the cell membrane, releasing mature exosomes into the extracellular space [34]. Microvesicles as well as apoptotic bodies are formed by outward budding of the plasma membrane. Additionally, EVs are critical in physiological processes such as immune response modulation, tissue repair and the development of cancer, due to their ability to transfer this bioactive cargo across biological barriers X [121].

EVs often vary in their size, composition and abundance depending on the type of cancer. Normally cancer cells produce a high amount of EVs in comparison to non-cancer cells [105]. The tumor microenvironment has been implicated in the high production on EVs by cancer cells by increasing hypoxia signaling [105], acidification [13] and inflammatory mechanisms [6]. Shibata et al. showed that low glucose levels drive pancreatic cancer cells to adjust their lipid composition, resulting in the secretion of smaller extracellular vesicles [93]. The composition of cancer extracellular vesicles (EVs) is as complex as the molecular landscape of cancer cells, binding functional molecules such as proteins, nucleic acids, and lipids, which together support their functional interactions within the cancer, the TME and between cells. Recent studies in cancer EVs showed the presence of cytosolic signaling proteins, cell surface receptors, transcription factors, metabolic enzymes, and extracellular matrix proteins. Furthermore, EVs are also enriched with RNA transcripts, microRNAs, and genomic DNA fragments (Fig. 3) [36]. EVs abundance also varies depending on the stage and the type of cancer reflecting the unique microenvironment of each cancer [28]. Some cancers are characterized by high levels of EVs secretion (such as pancreatic and ovarian cancer) facilitating tumor progression, metastasis, and immune evasion [28], while others cancer release lower levels still exert profound effects on tumorigenesis and intercellular communication.

Studies have increasingly focused on the role of EVs in liquid biopsy diagnostics [19,75]. EVs are largely used as cancer biomarkers in liquid biopsy samples. It was demonstrated that EV-bound Survivin (anti-apoptosis protein) was highly expressed in plasma collected from prostate [42] and breast [41] cancer patients compared to healthy persons, suggesting that EV-bound Survivin could serve as a potential diagnostic biomarker shared among multiple types of cancer. In 2019, a significant increase of AHSG (Alpha-2-HS-glycoprotein) and ECM1 (extracellular matrix protein 1) in NSCLC (Non-small-cell lung cancer) patients compared to healthy controls was reported [73]. These finding suggest that EVs-bound AHSG and ECM1 could also serve as biomarker for the diagnosis of NSCLC cancer. Other studies showed that exosomal circUHRF1 originates from hepatocellular carcinoma cells promote the immunosuppression by creating a high resistance against anti-PD1 immunotherapy and by inducing NK cell dysfunction, suggesting a prospective therapeutic avenue for HCC patients (P.-F [120]). As clinical and research studies on EVs continue to advance, using EVs as potential biomarkers has a great impact in the medical domain to improve management and cancer patients’ outcomes [18,26]

### Cell-free nucleic acids

Cell free nucleic acids (cfNAs) are fragments of DNA or RNA, which move freely in body fluids such as blood, urine or saliva. These nucleic acids are derived from cells under normal physiological processes and cellular damage or even death. cfNAs can be divided into cfDNA and cfRNA. cfDNA, in particular, has emerged as a promising biomarker for non-invasive detection and monitoring of various diseases, including cancer. cfRNA include coding RNA (mRNA) and non-coding RNA: long non-coding RNA, circular RNA, small RNA or miRNA (most studied cfRNA) [97]. Cell free nucleic acids allow early detection, monitoring of treatment responses and assessment for minimal residual diseases in patient’s cancer.

Recent studies showed that pancreatic and biliary tract cancers can be detected and characterized using liquid-biopsy-cfDNA fragmentomics X [92]. Luiz Fernando de Queiroz et al. also described a new method to monitor breast cancer patients by monitoring the methylation status of CDKN2A/p16INK4A (cyclin-dependent kinase inhibitor 2A) and RB1 (retinoblastoma transcriptional corepressor 1) using cfDNA. This analysis identified alterations in methylation even before any signs of breast cancer development were visible [81]. A Phase 1 study showed the potential role of plasma cfRNA as a novel biomarker to follow response to therapy with APG-157 in patients with head and neck cancer. Transcriptomic analysis indicated more leukocyte activation and more cytokines expression upon treatment with APG-157 in patients with head and neck cancer in comparison with health individuals [102].

While the use of liquid biopsy approaches for monitoring disease progression and identifying actionable mutations is well established, the use of liquid biopsy-based strategies to uncover tumor immune-evasion mechanisms in the immunotherapy setting to guide personalized treatment remains under active investigation. Circulating tumor DNA (ctDNA) and exosomal RNA can provide dynamic, non-invasive insights into tumor evolution and immune evasion pathways, such as acquired mutations in β2-microglobulin or JAK1/2, which are associated with resistance to immune checkpoint inhibitors [4,46]. Alterations in HLA expression and interferon signaling defects, which are increasingly linked to immune escape in patients undergoing ICI treatment, have been identified using liquid biopsy approaches [61].

Investigation into tumor immune-evasion mechanisms that result in ICI treatment failure require a biologically relevant context. For this purpose, the use of humanized mouse models—immunodeficient mice reconstituted with a human immune system—have emerged as a powerful preclinical platform to test new therapies that may improve ICI response. Xenograft models in humanized mice allow researchers to longitudinally track tumor-derived ctDNA mutations identified through liquid biopsy to monitor disease progression while simultaneously assessing treatment response and immune modulation in vivo [47,76]. As such, combining liquid biopsy with functional validation in humanized xenograft models enhances our ability to correlate molecular findings with therapeutic outcomes and provides a robust framework for investigating novel immunotherapy strategies. Although testing in a clinical setting is required, early evidence supports the potential utility of combining liquid biopsy with immune-profiling and model-based validation to tailor ICI regimens.

## Advancements in cancer immunotherapy studies using humanized mice models

The translation of mouse model findings to human studies has been limited by many genetic and biologic differences between humans and mice, some of which relate to immune function [32,66]. One meaningful way in which these constraints on cancer research are overcome is using humanized mice engrafted with a human immune system. Humanized mice models have bridged the gap between preclinical research and clinical applications in cancer immunotherapy. Novel models engrafted with human immune cells or tissues replicate the human immune response with fidelity in the murine host. This kind of fidelity allows for the examination of detailed in vivo interactions between the cancer cells and the human immune system, thus providing a solid platform for testing novel immunotherapies and discovering new biomarkers.

Several models of humanized mice have been developed for use in cancer immunotherapy studies, with NSG mice being the most commonly used mice in humanized models. NSG mice are derived from a strain called NOD (NOD/ShiLtJ) which has a severe combined immunodeficiency trait impairing T cell, B cells, and NK cells. PBMC—NSG mice are mice engrafted with human peripheral blood mononuclear (PBMC) cells, containing a wide range of immune cells, which are used to study the immune response toward tumors and also to test therapies modulating immunity H [122]. Patient-derived xenograft (PDX) models, which use tumors derived from human patients in mice with humanized immune system, are also used to recapitulate the human TME needed for personalized cancer immunotherapy studies [84,95]. CAR-T cell therapy, which uses T cells from a patient's body that are genetically engineered with chimeric antigen receptors that have the ability to selectively target specific antigens on cancer cells, are also tested using humanized mouse models Once reinfused into the patient, these newly engineered CAR-T cells can now recognize and kill the cancer cells, thus representing one of the most powerful and focussed immunotherapies against their form of cancer. Humanized mouse models are largely used to evaluate the efficacy and safety of CAR-T cell therapies against human tumors [22,89]. Humanized cytokine transgenic mice are another model genetically modified to induce the production of cytokines and they are used to study the roles of cytokines in cancer immunotherapy and to test new anti-cancer drugs [43]. Many other studies used the CD34+ hematopoietic stem cell (HSC) engrafted mice model transplanted with human CD34+ hematopoietic stem cells aiming to develop a complete human immune system, including B cells, T cells and myeloid cells. This mouse model is used to study ICI treatment and cancer immunotherapy. Ikumi Katano et al. used this mice model to study the anti‑cancer effects of anti‑PD‑1 antibody in several types of cancer and to predict how to improve this type of treatment [40]. Finally, humanized NSG-SGM3 mice engrafted with human CD34+ hematopoietic stem cells and transgenic for human cytokines IL-3, GM-CSF, and SCF also support development of a more robust human immune system and enhance studies related to cancer immunotherapy [50]; R. A [68]. In conclusion, these humanized mouse models allow the study of human immune responses against tumor cells, the efficacy of immunotherapies and mechanisms by which tumors evade from immune surveillance.

## Conclusion

Liquid biopsy is a non-invasive technique that enables the study of resistance mechanisms in tumors by analyzing the circulating genetic material, proteins, or tumor cells in blood or other body fluids. The precise identification of mutations or alterations in tumor DNA, RNA, or immune signatures helps to understand how tumor cells evade therapeutic strategies, including those that involve immune checkpoint inhibitors. This is important because it can highlight specific mechanisms of resistance that may give valuable insights into treatments tailored to the unique characteristics of a patient's tumor. Based on this information, personalized treatment strategies can be developed that aim at targeting the tumor more effectively while minimizing side effects. Moreover, these strategies can be evaluated in humanized mouse models that recapitulate the human immune system, enabling researchers to gauge how different treatments might perform in real patients. This approach holds great promise for the development of more effective and personalized therapies capable of overcoming resistance and ultimately improving clinical outcomes for cancer patients. Where the research is taken to the extent that liquid biopsy could provide not only monitoring treatment efficacy but also the detection of minimal residual disease and relapse earlier than conventional imaging techniques, it might revolutionize cancer management. Liquid biopsy offers a dynamic, real-time understanding of tumor evolution and immune response and thus enables a shift toward more adaptive and precise treatment regimens. Ultimately, its translation into the clinic could mean earlier interventions, improved prognosis for patients, and a substantial decrease in the burden of cancer treatment.

## CRediT authorship contribution statement

**Zeinab Dalloul:** Writing – original draft, Conceptualization. **Jana Grenel Briend:** Software, Resources. **Mariama Diawara:** Writing – review & editing. **Catherine Taylor:** Writing – review & editing, Conceptualization. **Rodney J. Ouellette:** Writing – review & editing, Supervision, Resources, Funding acquisition, Conceptualization.

## Declaration of competing interest

The authors declare that they have no known competing financial interests or personal relationships that could have appeared to influence the work reported in this paper.
